# 3D-Printed PCL/PPy Conductive Scaffolds as Three-Dimensional Porous Nerve Guide Conduits (NGCs) for Peripheral Nerve Injury Repair

**DOI:** 10.3389/fbioe.2019.00266

**Published:** 2019-10-16

**Authors:** Sanjairaj Vijayavenkataraman, Sathya Kannan, Tong Cao, Jerry Y. H. Fuh, Gopu Sriram, Wen Feng Lu

**Affiliations:** ^1^Department of Mechanical Engineering, National University of Singapore, Singapore, Singapore; ^2^Division of Engineering, New York University Abu Dhabi, Abu Dhabi, United Arab Emirates; ^3^Department of Mechanical Engineering, Tandon School of Engineering, New York University, New York, NY, United States; ^4^Faculty of Dentistry, National University of Singapore, Singapore, Singapore

**Keywords:** EHD-jet 3D printing, nerve guide conduit, tissue engineering scaffolds, conductive scaffolds, stem cells, peripheral nerve injury

## Abstract

Conductivity is a desirable property of an ideal nerve guide conduit (NGC) that is being considered for peripheral nerve regeneration. Most of the conductive polymers reported in use for fabrication of tissue engineering scaffolds such as polypyrrole (PPy), polyaniline, polythiophene, and poly(3,4-ethylenedioxythiophene) are non-biodegradable and possess weak mechanical properties to be fabricated into 3D structures. In this study, a biodegradable and conductive block copolymer of PPy and Polycaprolactone (PPy-b-PCL) was used to fabricate 3D porous NGCs using a novel electrohydrodynamic jet 3D printing process which offers superior control over fiber diameter, pore size, porosity, and fiber alignment. PCL/PPy scaffolds with three different concentrations of PPy-b-PCL (0.5, 1, and 2% v/v) were fabricated as a mesh (pore size 125 ± 15 μm) and the effect of incorporation of PPy-b-PCL on mechanical properties, biodegradability, and conductivity of the NGCs were studied. The mechanical properties of the scaffolds decreased with the addition of PPy-b-PCL which aided the ability to fabricate softer scaffolds that are closer to the properties of the native human peripheral nerve. With increasing concentrations of PPy-b-PCL, the scaffolds displayed a marked increase in conductivity (ranging from 0.28 to 1.15 mS/cm depending on concentration of PPy). Human embryonic stem cell-derived neural crest stem cells (hESC-NCSCs) were used to investigate the impact of PPy-b-PCL based conductive scaffolds on the growth and differentiation to peripheral neuronal cells. The hESC-NCSCs were able to attach and differentiate to peripheral neurons on PCL and PCL/PPy scaffolds, in particular the PCL/PPy (1% v/v) scaffolds supported higher growth of neural cells and a stronger maturation of hESC-NCSCs to peripheral neuronal cells. Overall, these results suggest that PPy-based conductive scaffolds have potential clinical value as cell-free or cell-laden NGCs for peripheral neuronal regeneration.

## Introduction

Scaffolds play an integral role in tissue engineering and regeneration by providing the structural support and mechanical cues for the adhesion, growth, proliferation, and differentiation of cells (O'brien, 2011; Vijayavenkataraman et al., 2017). Biocompatibility, biodegradability, porous, and biomimetic architecture are some of the essential properties of tissue engineering scaffolds (Zhang et al., 2019). In addition to these basic requirements, conductivity is a highly desirable property (Balint et al., 2014). Normal biological functions such as wound healing, muscle contraction, and nerve signaling are significantly influenced and controlled by the bioelectricity present in the human body (Ghasemi-Mobarakeh et al., 2011). Hence, providing a conductive scaffold would help in enhanced tissue regeneration by providing a connecting link for the normal bioelectric flow in the body. In addition, the cellular activities such as cell migration, DNA synthesis and protein secretion could be modulated by external electrical stimulation (Rouabhia et al., 2013; Wang et al., 2017).

Nerve Guide Conduits (NGCs) are being pursued as alternate method of treatment for peripheral nerve injuries (PNI), to overcome the shortcomings of autografts (Vijayavenkataraman et al., 2018). Conductivity plays a critical role in neuronal tissue regeneration (Ghasemi-Mobarakeh et al., 2011). Hence, in neural tissue engineering and neuronal regeneration treatment of neurodegenerative diseases such as Parkinson's disease, there is an emphasis on using conductive substrates and scaffolds to enhance the electrical conduction through the tissue engineering scaffolds to increase cell differentiation and tissue regeneration (Sirivisoot et al., 2014). The most commonly used conductive polymers in tissue engineering are polypyrrole (PPy), polyaniline (PANI), polythiophene (PTh), and poly(3,4-ethylenedioxythiophene) (PEDOT) (Balint et al., 2014). Most of the biological studies involving conductive polymers are performed on 2D substrates (Jakubiec et al., 1998; Gumus et al., 2010; Ghasemi-Mobarakeh et al., 2011). However, it is a well-known and well-established fact that the three-dimensional (3D) microenvironment mimics the natural tissue conditions in the body more closely than traditional monolayer cultures (Cukierman et al., 2001; Pampaloni et al., 2007). Conductive polymers possess weak mechanical properties and poor processability to be fabricated into 3D scaffolds (Lee, 2013). Conductive polymers can be mixed or copolymerized along with other polymers such as polycaprolactone (PCL) to enhance the mechanical and rheological properties and thereby its processability into 3D scaffolds.

There are previous works reported on conductive electrospun scaffolds for tissue engineering of neural, cardiac, and skeletal muscles, where a conducting polymer is mixed with other electrospinnable polymers are fabricated into nanofibrous scaffolds. Some of these works include electrospinning of PPy-Polyethylene Oxide (PEO) (Chronakis et al., 2006), PANI-gelatin (Li et al., 2006), PANI-Polycaprolactone (PCL) (Chen et al., 2013), PANI-PCL-gelatin (Ghasemi-Mobarakeh et al., 2009), and PPy-Poly(lactic acid) (PLA) (Zong et al., 2005). In these studies, the conductive scaffolds were found beneficial to the neural cell proliferation, growth, and differentiation, however, there are a few limitations. Firstly, the conductive polymers that were used are non-biodegradable. Biodegradability is an important prerequisite of any tissue engineering scaffold. Secondly, the limitations of the electrospinning process in terms of less control over the micro- and macro-architecture of the scaffolds (Jaworek and Krupa, 1999). The pore size, porosity, and fiber direction cannot be controlled precisely in the electrospinning process.

**Figure 1:**
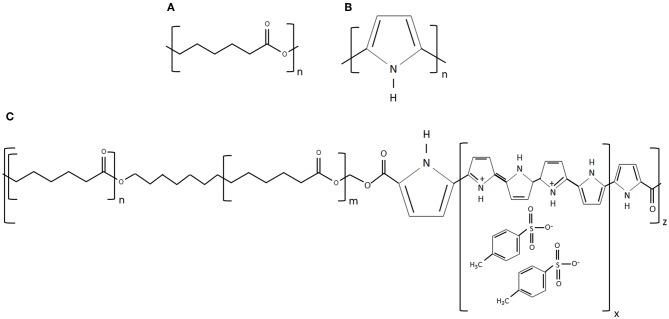
**(A)** Chemical structure of PCL, **(B)** chemical structure of PPy, and **(C)** chemical structure of PPy-b-PCL (chemical structures reproduced from www.sigmaaldrich.com).

To overcome the above limitations, in this work, we used a novel 3D printing process called electrohydrodynamic jet (EHD-jet) 3D printing is used to print PCL-based scaffolds (Liu et al., 2017; Wu et al., 2017), which offers a superior control over pore size, porosity, fiber diameter, and alignment. Furthermore, to enable conductive properties to the scaffolds, PCL was mixed with a conductive block copolymer of PPy and PCL (PPy-b-PCL) (shown in Figure 1). Three different concentrations of PPy-b-PCL (0.5, 1, and 2% v/v) are mixed with PCL (70% w/v) in glacial acetic acid and fabricated into 3D porous scaffolds. To mimic the tubular nature of NGCs, the sheets of 3D printed scaffolds were rolled into tubular conduits and heat-sealed, and the material, mechanical and biodegradation properties are characterized. As a proof-of-concept for the application of these PCL/PPy-based conductive scaffolds as NGCs for peripheral nerve regeneration, *in vitro* neural differentiation studies were carried out using human embryonic stem cells -derived neural crest stem cells (hESC-NCSCs).

## Experimental Section

### Materials

Polycaprolactone (PCL) pellets (80 kDa), Polypyrrole-block-poly(caprolactone) (PPy-b-PCL) and glacial acetic acid (>99.7% pure) were purchased from Sigma-Aldrich Pte Ltd., Singapore.

### Preparation of PCL and PCL/PPy Solution and Scaffold Fabrication

A concentration of 70% (w/v) PCL in acetic acid is was prepared by ultra-sonication at 60°C and 40 kHz for 3 h. For PCL/PPy solution, three different concentrations of PPy-b-PCL (0.5, 1 and 2% v/v) were mixed with acetic acid, PCL pellets (70% w/v) were then added into the PPy-b-PCL/acetic acid solution and was ultra-sonicated at the same conditions. The solutions prepared are then fabricated into 3D scaffolds using an in-house built EHD-jet 3D printing system, the specifications of the system were published in our previous works (Vijayavenkataraman et al., 2018).

### Material Characterization

#### Scanning Electron Microscope, Raman Spectroscopy, and Wettability

Scaffolds were imaged using a scanning electron microscope (JEOL JSM-5500) and an image analysis software (ImageJ, National Institute of Health, Bethesda, MD) was used to calculate the average pore size and fiber diameter. Horiba Jobin Yvon Modular Raman Spectrometer at a laser excitation wavelength of 514 nm (Stellar Pro Argon-ion laser) was used to record the Raman spectra. VAC Optima Surface Analysis System (AST Products, Billerica, MA) was used to measure the contact angle.

#### Differential Scanning Calorimetry (DSC), Thermo-Gravimetric Analysis (TGA), and Differential Thermal Analysis (DTA)

DSC and TGA of scaffolds (~1 mg each) was performed using a differential scanning calorimeter (Perkin-Elmer Diamond DSC) and a thermogravimetric analyzer (Perkin Elmer Pyris 1), respectively, at a heating rate of 10°C/min (Argon atmosphere).

#### Conductivity

A conductivity meter (SevenCompactTM pH/Ion meter S220, Mettler-Toledo Singapore Pte Ltd., Singapore) was used to measure the conductivity of PCL/PPy-b-PCL solutions.

### Mechanical Testing

Mechanical properties of tubular NGCs and rectangular scaffold samples (in degradation studies) were obtained from tensile testing (Instron 3345, USA, 100 N load cell) at 10 mm/min strain rate.

### Degradation Studies

The scaffold samples (30 mm long and 5 mm wide) with the initial weight (W_i_) were submerged in 10 mL of 0.5 M NaOH solution at a pH of 13.36 and maintained at 37°C in an incubator with shaker to mimic the physiological conditions. One set (*n* = 3) of samples was removed at each time point, dried at room temperature for 48 h, which are then weighed (W_dry_) and tested for their mechanical properties (refer section Mechanical Testing). Gravimetric analysis was performed to determine the weight loss at each time point using Equation (1).

(1) $$\begin{array}{r} \text{Weight}\;\text{loss}\;\left(\%\right)=\left({\text{W}}_{\text{i}}-{\text{W}}_{\text{dry}}\right)/{\text{W}}_{\text{i}}\times 100\%. \end{array}$$

### Neural Crest Stem Cell *in vitro* Studies

#### Cell Culture

hESC-NCSCs were used for the cell culture studies. The detailed protocols for obtaining NCSCs from hESCs and differentiation of NCSCs to peripheral neurons are published previously (Zhu et al., 2017). PCL/PPy scaffolds were cut in shape to fit the 24-well plate. The scaffolds were soaked in 70% ethanol for 30 min, and then rinsed twice with phosphate buffered saline (PBS), and DMEM/F12 (Life Technologies). To aid cell attachment (as the scaffolds are relatively hydrophobic), they were coated with Matrigel (BD Biosciences) (10 μg/ml) in DMEM/F12 for 2 h prior to cell seeding at a density of 25,000 cells per cm^2^, and were placed in ultralow attachment cell culture plates. Culture media consisted of neurobasal media (Life Technologies) supplemented with 1 × non-essential amino acids, 1 × GlutaMAX™ (Sigma), 1 × N2, 1 × B27 (Life Technologies), 20 ng/ml EGF (Sigma), 20 ng/ml bFGF, 10 ng/ml nerve growth factor-β, and 25 μM Y27632 (Miltenyi Biotec) for differentiation of hESC-derived NCSCs to peripheral neurons. Media changes were performed once every 3 days for 2 weeks, after which the differentiated peripheral neurons were analyzed for protein expression by immunocytochemistry.

#### Cell Proliferation Assay

Cell proliferation was investigated by performing MTS assay (CellTiter 96 AQueous One Solution Cell Proliferation Assay, Promega) at 3 and 7 days. Cells were seeded as mentioned above onto the PCL/PCLPPy scaffolds placed within ultralow attachment 24-well plates. Cells were washed with 1 × PBS and incubated with CellTiter 96^®^ AQueous One Solution in DMEM for 1 h at 37°C. After incubation the supernatant was removed to a 96-well plate and the absorbance was measured at 490 nm with Infinite 200 microplate reader (Tecan). Negative control included CellTiter 96 AQueous One Solution in DMEM without cells.

#### RT-PCR

The RNA from cultured cells were isolated using MN NucleoSpin RNA kit (Macherey-Nagel, Germany) according to the manufacturer's instructions. Nanodrop ND-1000 spectrophotometer (Nanodrop technologies, Wilmington, DE) was used to quantify the extracted RNA. 1 μg of mRNA was reverse transcribed into cDNA using iScript™ cDNA Synthesis Kit (BioRad) in the MyCycler™ Thermal Cycler System (BioRad). Real-time PCR was performed in triplicates with 500 ng cDNA template per reaction using iTaq™ Universal SYBR Green Supermix (BioRad) and CFX Connect™ Real-Time System (BioRad) as per manufacturer's instructions. The sequences of the forward and reverse primers of genes analyzed are provided in Supplementary Table 1. The target gene expression was normalized to GAPDH as the internal control, and results were expressed as fold change relative to cells grown on control PCL scaffolds.

#### Immunocytochemistry and Image Analysis

Cells were fixed with 4% paraformaldehyde (Sigma) for 30 min at room temperature. Fixed cells were permeabilized using 0.1% TritonX-100/PBS (Sigma) for 10 min, washed thrice with 0.05% Tween-20/PBS (Sigma), and blocked with 2% bovine serum albumin/5% goat serum for 60 min to prevent non-specific binding. Subsequently, the cells were labeled with β3 tubulin [1:200, Santa Cruz sc-58888, monoclonal (Clone TuJ-1)] and neurofilament heavy (NF-H) (1:600, Abcam ab8135, polyclonal) primary antibodies at 4°C overnight. After washing, the cells were fluorescently labeled using appropriate secondary antibodies Alexa Flour 488 and Alexa Flour 594 (Life Technologies) for 60 min, then counterstained with 4′,6-diamidino-2-phenylindole (DAPI, Sigma). Images were taken with Olympus FV1000 confocal microscope. Imaris software was used to quantify the protein markers β3 tubulin and NF-H. From the immunostained 3D z-stack images, mean fluorescent intensity values of β3 tubulin (green channel) and NF-H (red channel) were measured, from which the fluorescence was normalized to β3 tubulin expression to obtain the normalized fluorescence intensity. Then, total volume of expression for these two proteins were calculated; data was normalized to the total cell numbers (based on counting DAPI stained nuclei) present in each region of interest to get the level of expression of the two proteins. Detailed information provided in supplementary information (Supplementary Figure 1 and Supplementary Materials).

### Statistical Analysis

Experiments were run in triplicates and all measurements were expressed as mean ± SD. One-way ANOVA test and Independent 2-sample *t*-test were used to determine the differences between the mean values of the experimental groups Differences (statistically significant at *p* < 0.05).

## Results

### Impact of PCL-b-PPy on Scaffold Surface Characteristics

**Figure 2:**
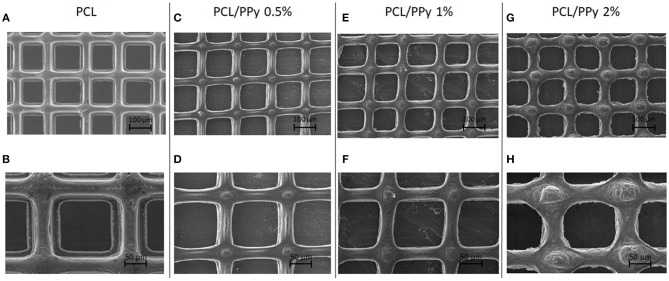
SEM images of EHD-jet 3D-printed scaffolds for nerve guide conduits (NGCs) (input voltage = 2.4 kV, stage speed = 75 mm/min, flow rate = 10 μl/min, nozzle-to-substrate distance = 2 mm) **(A,B)** PCL scaffolds, **(C,D)** PCL/PPy 0.5%, **(E,F)** PCL/PPy 1%, and **(G,H)** PCL/PPy 2% at different magnifications.

Figures 2A–H shows the SEM images of PCL (Figures 2A,B), PCL/PPy 0.5% (Figures 2C,D), PCL/PPy 1% (Figures 2E,F), and PCL/PPy 2% (Figures 2G,H) scaffolds. The average fiber diameters measured are 30 ± 10 μm (PCL), 33 ± 8 μm (PCL/PPy 0.5%), 38 ± 7 μm (PCL/PPy 1%), and 44 ± 5 μm (PCL/PPy 2%), with an average pore size of 125 ± 15 μm. The addition of PPy-b-PCL changes the scaffold surface texture, with rigid fibers and sharp edges in PCL scaffolds and blunt fibers and rounded edges in PCL/PPy scaffolds (more rounded with increasing PPy-b-PCL) as visible in PCL/PPy 2% in Figures 2G,H.

### Impact of PCL-b-PPy on Material Composition of PCL Scaffolds

**Figure 3:**
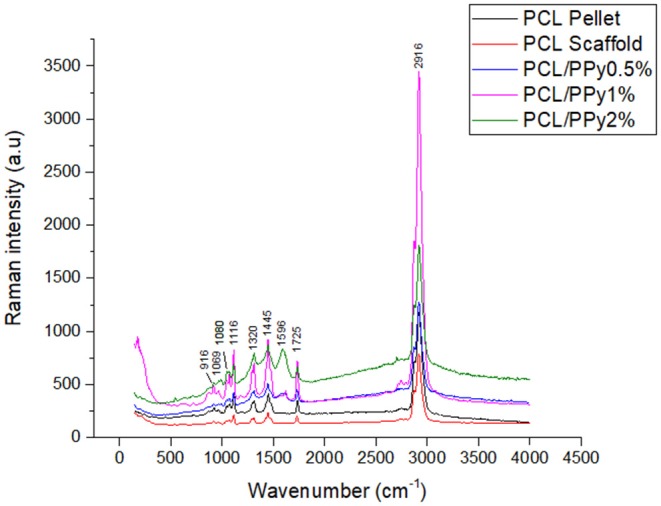
Raman spectra of as-received PCL pellets, and EHD-jetted scaffolds.

The Raman spectrum of the EHD-jetted scaffolds is shown in Figure 3, the band positions and assignments (Vigmond et al., 1995; Chen et al., 2003) are tabulated in Table 1. The characteristic peaks of PCL (1,725, 2,916, 1,116, 916 cm^−1^) are present in both as-received PCL pellets and EHD-jetted scaffolds suggesting that the EHD-jetting process did not alter the material composition of PCL. The characteristic peaks of PPy, namely C-H in-plane deformation at 1,080 cm^−1^, C-H ring stretching at 1,320 cm^−1^, and C = C symmetrical stretching at 1,596 cm^−1^ are present in the PCL/PPy scaffolds but are not present in as-received PCL pellets and PCL scaffolds, confirming the presence of PPy in the PCL/PPy scaffolds. In summary, the spectrum suggests that there are no major changes in the material composition due to the fabrication process.

**Table 1:** Raman bands [cm^−1^] and their assignments for EHD-jetted PCL/PPy Scaffolds (Vigmond et al., 1995; Chen et al., 2003).

**Raman bands (cm^**−1**^)**	**Assignments**	
916	PCL	ν(C–COO); crystalline
1,069	PCL	ν(COC); crystalline
1,080	PPy	C-H in-plane deformation
1,116	PCL	ν(COC); crystalline
1,320	PPy	C-H ring stretching
1,445	PCL	δ(CH_2_); crystalline
1,596	PPy	C = C symmetrical stretching
1,725	PCL/PPy	ν(C = O); crystalline
2,916	PCL	Antisymmetric C–H stretching ν(CH_2_)_asym_

### Addition of PCL-b-PPy Has Minimal Impact on Hydrophilicity of PCL Scaffolds

**Figure 4:**
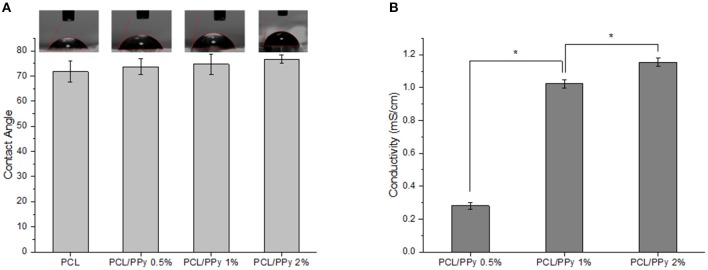
Hydrophilicity and conductivity properties of PCL and PCL/PPy scaffolds **(A)** water contact angle, and **(B)** conductivity of 3D-printed scaffolds (*n* = 3; ^*^p < 0.05).

The wettability of EHD-jetted scaffolds are measured to be 71.7 ± 4.1° (PCL), 73.7 ± 3.1° (PCL/PPy 0.5%), 74.7 ± 3.9° (PCL/PPy 1%), and 76.6 ± 1.7° (PCL/PPy 2%) as shown in Figure 4A. There was no significant changes in the wettability of the scaffolds with the addition of PPy-b-PCL, with only a slight increase of water contact angle due to the hydrophobicity of PPy.

### Inclusion of PCL-b-PPy Dramatically Increases the Conductivity of PCL Scaffolds

Conductivity measurements were recorded as 0.09 ± 0.005 μS/cm (PCL) (insignificant, mainly due to acetic acid), 0.28 ± 0.02 mS/cm (PCL/PPy 0.5%), 1.02 ± 0.03 mS/cm (PCL/PPy 1%), and 1.15 ± 0.03 mS/cm (PCL/PPy 2%). There is significant increase in the conductivity with increasing concentration of PPy-b-PCL, as shown in the Figure 4B.

### Impact of PCL-b-PPy on Thermal Properties of PCL Scaffolds

**Figure 5:**
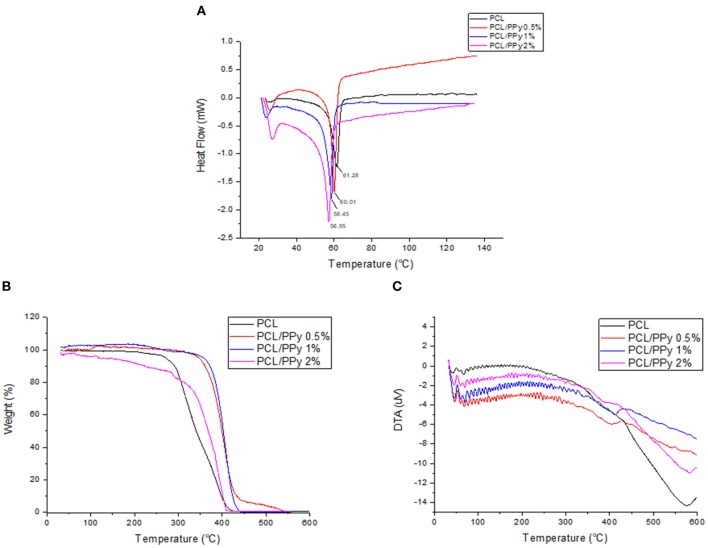
Thermal degradation of PCL and PCL/PPy scaffolds: **(A)** DSC Thermogram, **(B)** TGA curves, and **(C)** DTA curves of EHD-jetted scaffolds.

DSC thermograms, representing the thermal properties of END-jetted scaffolds are shown in Figure 5A. The melting point of PCL scaffolds was within 57–64°C range, implied by the endothermic peak observed at 61.28°C (Acharya et al., 2012). The endothermic peak shifted to lower temperatures with addition of PPy-b-PCL (60.01°C for PCL/PPy 0.5%, 58.45°C PCL/PPy 1%, and 56.95°C PCL/PPy 2% scaffolds), indicating PPy-b-PCL presence, which agrees with the previous studies (Lu et al., 2010). Also, it is known from the literature that the endothermic peak (50°C) of pure PPy increased in intensity when grafted with PCL (Mecerreyes et al., 2002). TGA and DTA curves were shown in Figures 5B,C, respectively. The onset decomposition temperatures of PCL scaffold is around 300°C (as shown in Figure 5B), which is in agreement with the earlier studies (Liu et al., 2013). The onset decomposition temperature of PCL/PPy 0.5%, PCL/PPy 1%, and PCL/PPy 2% scaffolds were greater than that of the PCL scaffold (between 350 and 450°C), indicating the presence of PPy-b-PCL (Ramaprasad et al., 2017). A two-step decomposition process could be observed in all the scaffolds. The first decomposition step for PCL occurs between 50 and 300°C (Arunraj et al., 2013) and for PCL/PPy scaffolds between 50 and 350°C (Najar and Majid, 2014; Ramaprasad et al., 2017). The first step of decomposition is attributed to residual water loss. The second decomposition step for PCL occurs between 300 and 450°C (Arunraj et al., 2013) and for PCL/PPy scaffolds between 350 and 500°C (Najar and Majid, 2014; Ramaprasad et al., 2017). The second step of decomposition is due to the breakdown of the polymer chain, mainly the polysaccharide structure of the molecule (Arunraj et al., 2013). DTA curves (Figure 5C) shows smaller endothermic peaks between 50 and 350°C, and exothermic peaks between 350 and 450°C, indicating absorbed water loss and continuous polymer chain decomposition, respectively (Liu et al., 2013; Ramaprasad et al., 2017). The DSC and TGA curves of PCL/PPy scaffolds follows the same characteristics as that of pure PCL scaffolds, suggesting that the thermal properties was not significantly altered with PPy-b-PCL addition. PPy-b-PCL renders the conductive property to the scaffolds. Since PCL is a widely used biomaterial and used for *in vivo* experiments already, PCL/PPy scaffolds is also inferred to be safer as the properties are very similar to PCL scaffolds. The DSC and TGA curves also suggest the biodegradability of the EHD-jetted PCL/PPy scaffolds.

### Incorporation of PCL-b-PPy Aids Fabrication of Softer Scaffolds Suitable for Neural Applications

**Figure 6:**
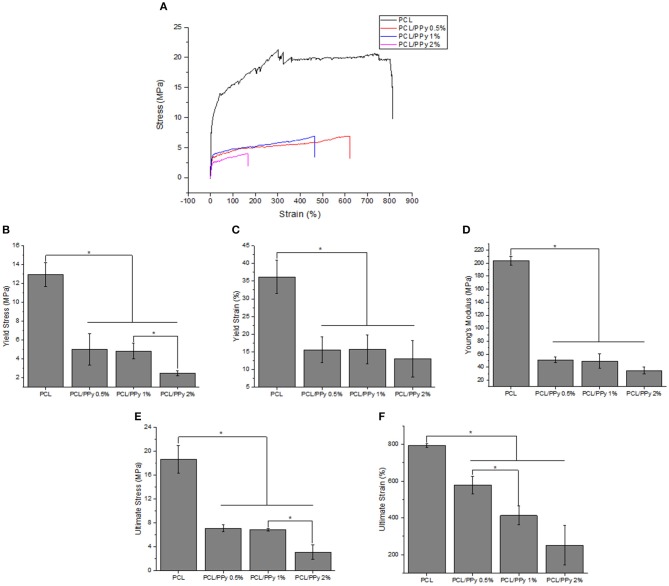
Mechanical properties of PCL and PCL/PPy NGCs: **(A)** representative stress-strain curves **(B)** Young's modulus, **(C)** yield stress, **(D)** yield strain, **(E)** ultimate stress, and **(F)** ultimate strain (*n* = 3, ^*^p < 0.05).

The stress-strain curves and the mechanical properties of NGCs are shown in Figures 6A–F, respectively. A significant reduction of Young's modulus of the NGC structure was observed between PCL (204 ± 6.7 MPa) and PCL/PPy scaffolds (35 ± 5.6 MPa with PCL/PPy 2%). However, between PCL/PPy 0.5%, PCL/PPy 1%, and PCL/PPy 2%, there is not much difference, with the range of Young's modulus between 51 ± 4.55 MPa (PCL/PPy 0.5%) and 35 ± 5.6 MPa (PCL/PPy 2%). Similar trend is observed with the yield strength and ultimate strength. This demonstrated decrease in the mechanical properties with the addition of PPy-b-PCL is indeed a desired effect as softer scaffolds aid in better neural cell differentiation (Vijayavenkataraman et al., 2017).

### Incorporation of PCL-b-PPy Improves Degradation Properties of PCL Scaffolds

**Figure 7:**
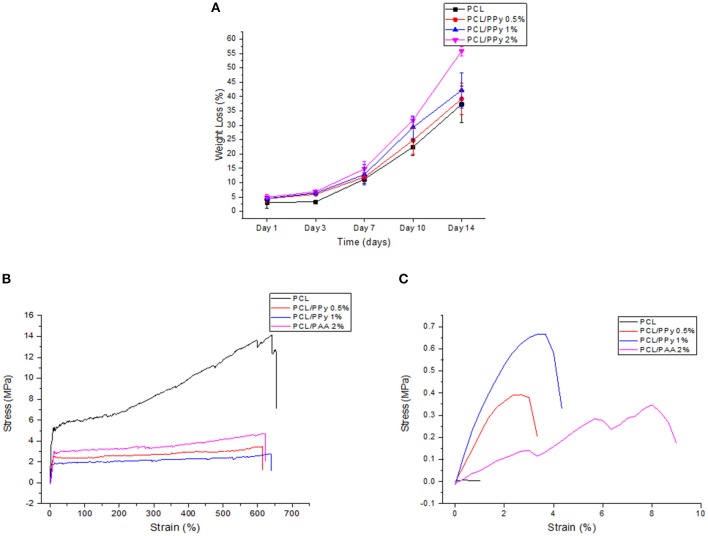
Accelerated degradation studies on PCL and PCL/PPy scaffolds: **(A)** percentage of weight loss as a function of degradation time, **(B)** representative stress-strain curves at day 0, and **(C)** day 14.

The weight loss behavior and representative stress-strain curves (on Days 0 and 14) of the 3D-printed scaffolds are shown in Figures 7A–C, respectively.

The scaffold degradation rate increased with increasing PPy-b-PCL concentration (Figure 7A), with 55.8% weight loss in PCL/PPy 2% scaffolds against 37.28% in PCL scaffolds at day 14. The pH values of the immersed medium decreased from an initial pH of 13.36–13, for all the four different scaffold types. Figures 7B,C shows that all the scaffolds exhibited a typical polymeric stress-strain curve, but for the PCL/PPy 2% scaffolds, all of them lost most of their mechanical strength, corroborating to the weight loss trend.

The mechanical properties evaluated during the scaffold degradation studies are shown in Table 2. Better mechanical properties were observed with the rolled NGC structure shown in Figure 6 than that of the as-printed scaffolds in the degradation study (Table 2), the aim of which was to assess the influence of PPy-b-PCL addition on degradation more accurately. From the degradation studies, it can be deduced that the mechanical properties decrease as the weight decreases and attains a minimum after 3 days. From day 7, the scaffolds start disintegrating and the mechanical properties couldn't be determined, the stress-strain curves don't represent the characteristic stress-strain curve of typical polymers.

**Table 2:** Mechanical properties of PCL, PCL/PPy 0.5%, PCL/PPy 1%, and PCL/PPy 2% scaffolds at various degradation time points (X denotes indeterminable values).

**Mechanical property**	**Scaffold type**	**Day 0**	**Day 1**	**Day 3**	**Day 7**	**Day 10**	**Day 14**
Yield strength	PCL	4.57 ± 0.3	1.2 ± 0.3	1.01 ± 0.5	X	X	X
	PCL/PPy 0.5%	2.38 ± 0.1	1.47 ± 0.02	1.13 ± 0.03	0.57 ± 0.04	X	X
	PCL/PPy 1%	2.19 ± 1.6	3.31 ± 0.6	1.11 ± 0.2	0.82 ± 0.01	0.61 ± 0.02	X
	PCL/PPy 2%	3.05 ± 0.8	1.85 ± 0.8	1.7 ± 0.5	1.21 ± 0.1	0.42 ± 0.1	0.14 ± 0.02
Yield strain	PCL	27.45 ± 2.5	7.17 ± 2.6	4.05 ± 1.6	X	X	X
	PCL/PPy 0.5%	15 ± 2.4	10.56 ± 1.5	8.17 ± 3.5	2.51 ± 0.2	X	X
	PCL/PPy 1%	16.23 ± 2.8	5.89 ± 1.3	2.84 ± 0.7	1.23 ± 0.5	0.58 ± 0.3	X
	PCL/PPy 2%	11.34 ± 0.9	6.34 ± 1.9	5.78 ± 1.5	4.23 ± 0.5	3.21 ± 0.3	3 ± 0.2
Young's modulus	PCL	96.73 ± 4.4	58.63 ± 6.2	42.37 ± 7.5	X	X	X
	PCL/PPy 0.5%	42.97 ± 3.4	28.39 ± 5.7	19.27 ± 3.4	22.53 ± 1.5	X	X
	PCL/PPy 1%	41.12 ± 8.4	29.21 ± 3.2	17.89 ± 5.1	11.95 ± 2.5	2.53 ± 1.1	X
	PCL/PPy 2%	21.08 ± 3.9	20.24 ± 4.4	12.67 ± 2.3	9.42 ± 1.3	5.23 ± 1.8	5.58 ± 2
Ultimate strength	PCL	13.46 ± 1.6	1.68 ± 0.4	1.24 ± 0.9	X	X	X
	PCL/PPy 0.5%	4.4 ± 0.9	3.41 ± 0.2	1.2 ± 0.4	0.73 ± 0.2	X	X
	PCL/PPy 1%	3.26 ± 1.9	3.36 ± 0.5	1.3 ± 0.2	1.43 ± 0.4	0.93 ± 0.3	X
	PCL/PPy 2%	3.15 ± 1.4	2.38 ± 1.5	1.31 ± 0.3	0.85 ± 0.05	0.49 ± 0.02	0.35 ± 0.03
Ultimate strain	PCL	652.19 ± 45	281.75 ± 34	35.75 ± 6.5	X	X	X
	PCL/PPy 0.5%	607.91 ± 11	138.37 ± 22	17 ± 9.9	5.17 ± 1.2	X	X
	PCL/PPy 1%	569.43 ± 26	227.46 ± 13	19.25 ± 12	5.67 ± 0.8	4.23 ± 0.1	X
	PCL/PPy 2%	610.39 ± 12	208.17 ± 8	16.15 ± 6	10.56 ± 5	8.18 ± 0.5	8 ± 0.05

### PCL/PPy (1%) Scaffolds Support Proliferation and Stronger Maturation of NCSCs to Peripheral Neurons

**Figure 8:**
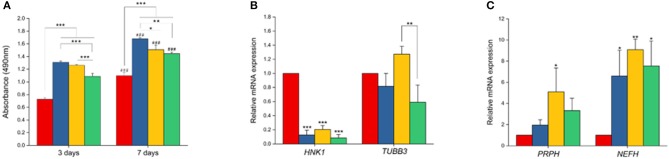
Cell proliferation and gene expression analysis. **(A)** MTS assay. Proliferation of NCSCs were tested at two different time points (3 and 7 days). **(B,C)** Gene expression levels of *HNK1, TUBB3, PRPH*, and *NEFH* by RT-PCR (*n* = 3 for all experiments). ^*^p < 0.05, ^**^p < 0.01, ^***^p < 0.001. ^###^in **(A)** refers to statistical significance (*p* < 0.001) when compared to first time-point (day-3) using independent Student *t*-test.

Cell culture on all scaffolds were performed to test if they supported proliferation and differentiation of neural lineage cell types. Here we observed the attachment of hESC-NCSCs to matrigel-coated scaffolds within the first 24 h of cell seeding. To assess the effect of PPy on cell proliferation, MTS assay was performed. Results showed significantly higher proliferation of hESC-NCSCs in all scaffolds containing PPy at days 3 and 7 when compared to pure PCL scaffolds (Figure 8A). Addition of PPy 0.5% showed the highest cell proliferation at both time points. Besides, there was a significantly higher proliferation of the hESC-NCSCs between days 3 and 7 in all the scaffolds demonstrating cyo-compatibility over long-term culture. Therefore, putting the results together addition of PPy to PCL scaffolds significantly increased the proliferation of hESC-NCSCs over the 7-day culture period.

**Figure 9:**
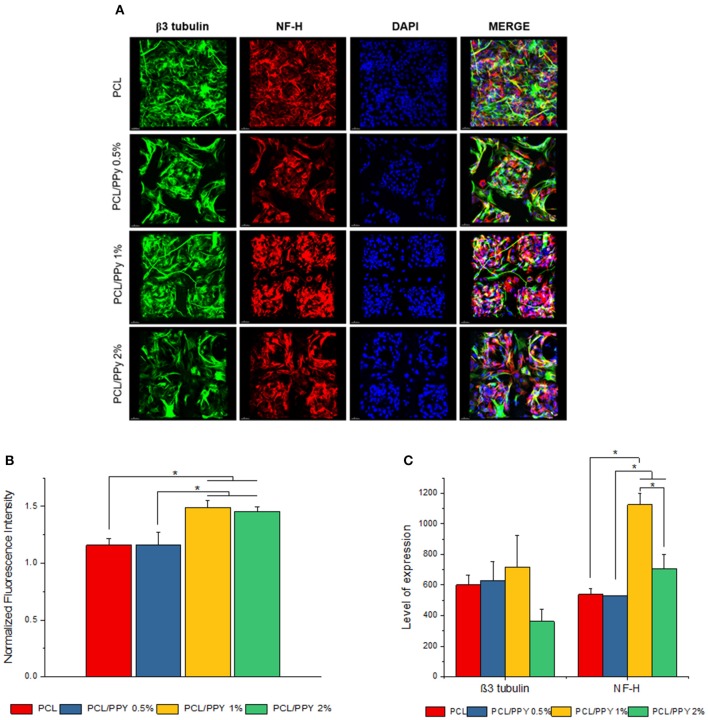
Expression of pan-neuronal marker (β3 tubulin) and peripheral neuron marker (NF-H) by immunocytochemistry. **(A)** 3D projection of the confocal z-stack images of hESC-NCSCs differentiated into peripheral neurons on PCL and PCL/PPy (0.5, 1, and 2%) scaffolds at day 14. Scale bar 30 μm. **(B)** Normalized fluorescence intensity of cells calculated from mean fluorescence intensity of NF-H (red channel) normalized to mean fluorescence intensity of β3 tubulin (green channel). **(C)** Volumetric fluorescence intensity of β3 tubulin and NF-H calculated from volume of expression relative to cell numbers (*n* = 3 for all experiments). ^*^p < 0.05.

Impact of PCL/PPy on the differentiation of hESC-NCSCs was assessed by RT-PCR and immunocytochemistry. Gene expression analysis by RT-PCR showed significant downregulation of transcripts for NCSC marker (*HNK1*) in the cells grown on all PCL/PPy scaffolds compared to PCL scaffolds (Figure 8B). Secondly, there was not much difference in the expression of *TUBB3* (β3 tubulin, a pan-neuronal marker), except between PCL/PPy1% and PCL/PPy2% (Figure 8C). Down-regulation of *HNK1* expression suggest that PCL/PPy aids differentiation of NCSCs, while the lack of difference in *TUBB3* expression among the different scaffolds suggest that the scaffolds aid growth of neural-lineage cells in general. This was further confirmed by the analysis of β3 tubulin by immunocytochemistry (Figures 9A,B). Assessment of volumetric fluorescence intensity of β3 tubulin staining showed no significant difference among the different scaffolds suggesting all the scaffold types offer a similar support to neural-lineage cell types (Figure 9C).

To specifically assess the differentiation of hESC-NCSCs to peripheral neurons, the expression of transcripts for *PRPH* (Peripherin, a peripheral neuron-specific marker) and *NEFH* (neurofilament-heavy subunit, NF-H; a peripheral neuron maturation marker) was assessed by RT-PCR. The results showed a trend toward higher expression of *PRPH* in PCL/PPy scaffolds, though not statistically significant except PCL/PPY1% scaffold. Additionally, the expression of *NEFH* transcripts, was significantly higher in cells differentiated on all PCL/PPy scaffolds when compared to pure PCL scaffolds (Figures 8B,C). These results suggest that PCL/PPy scaffolds potentially aid the differentiation of hESC-NCSCs toward peripheral neurons and in particular support their maturation. This was further confirmed by semi-quantitative assessment of the expression of the protein NF-H by immunocytochemistry (detailed methods for the semi-quantitative assessment are provided in the Supplementary Information). Normalized fluorescence intensity of NF-H (normalized to respective β3 tubulin) shows a significantly higher intensity of signals in the PCL/PPy (1 and 2%) scaffolds (Figure 9B). Secondly, assessment of volumetric fluorescence intensity of NF-H staining showed significantly higher intensity of signals in the PCL/PPy1% scaffolds. Collectively, these results indicate higher amounts of NF-H expressing mature peripheral neurons in PCL/PPy scaffolds (in particular PCL/PPy1%), Hence, the PCL/PPy scaffolds support differentiation and stronger maturation of peripheral neurons.

## Discussion

Conductivity is a desired property of NGCs which would aid in enhanced electrical conduction thereby increasing the cell differentiation and tissue regeneration (Sirivisoot et al., 2014). Though there are previous reports on electrospinning of conductive nanofibrous scaffolds (Prabhakaran et al., 2011; Sharma et al., 2012), they suffer from 2-fold limitation namely non-biodegradability and inability to control the scaffold morphology precisely. In this study, biodegradable PCL/PPy composites EHD-jetted into 3D porous NGCs (Vijayavenkataraman et al., 2019a,b). The effect of PPy-b-PCL concentration (0.5, 1, and 2%) on the mechanical properties of the NGCs are studied, with pure PCL scaffolds as the control. Our results demonstrated decrease in the mechanical properties with the addition of PPy-b-PCL, which is indeed a desired effect as softer scaffolds aid in better neural cell differentiation (Vijayavenkataraman et al., 2017). Accelerated *in vitro* degradation studies in 0.5 M NaOH solution at a pH of 13.6 demonstrated the degradability of PCL/PPy scaffolds. The degradation rate of PCL/PPy scaffolds are higher compared to the pure PCL scaffolds; the mechanical properties deteriorated with time and loses most of its mechanical strength after day 10.

NGCs fabricated from PCL and PPy-b-PCL has many advantages. Previous studies on conductive scaffolds use non-biodegradable conductive polymers such as PPy, PANI or PEDOT. In this study, a block copolymer of PPy and PCL (PPy-b-PCL) was used which is biocompatible and biodegradable at the same time (Boutry et al., 2013). PCL is an FDA approved polymer, easy to print and better mechanical stability (Middleton and Tipton, 2000). Ideally, NGCs must provide good structural, mechanical, and conductive support for they act as templates for axonal regeneration (Lackington et al., 2017). PCL provides the structural support and mechanical cues while PPy-b-PCL provides conductive properties that can be exploited for neuronal regeneration.

In this study, we used EHD-jet 3D printing process to print PCL and PCL/PPy (0.5, 1, and 2%) scaffolds, with 125 ± 15 μm pores. In another study of ours using PCL scaffolds with five different pore sizes (ranging from 125 to 550 μm) and *in vitro* neural differentiation of PC12 cells, a pore size of 125 ± 15 μm best supported neural differentiation (Vijayavenkataraman et al., 2018). The sheets of scaffolds were rolled to form NGCs, mimicking the native peripheral nerve dimensions (1.2 mm diameter, ~200 um wall thickness, 1–3 cm long), porosity > 60% (Chiono and Tonda-Turo, 2015) and with aligned fibers that serve as a directional cue for directing the growth and alignment of neurons. The fibers are directionally aligned and the pore size are precisely controlled in the EHD-jet 3D printing unlike the electrospinning process. Addition of PPy-b-PCL has only a minor influence on the scaffold surface morphology. The PCL/PPy scaffold surface is non-uniform and wavy in nature, which could be attributed to the viscosity changes due to addition of PPy-b-PCL. Raman spectra (Figure 3) of PCL/PPy scaffolds suggest that there are no major changes in the material composition due to the fabrication process. The wettability of PCL/PPy scaffolds does not differ significantly from that of pure PCL scaffolds (Figure 4A), while the conductivity increases significantly with addition of PPy-b-PCL (Figure 4B). The DSC and TGA curves indirectly demonstrates the biodegradability of the EHD-jetted PCL/PPy scaffolds and the thermograms of PCL/PPy scaffolds are characteristically similar to that of pure PCL scaffolds.

The NGC mechanical properties decrease with the addition of PPy-b-PCL, with insignificant difference PCL/PPy 0.5% and PCL/PPy 1% scaffold mechanical properties. The yield strength of the NGC decreases from 12.95 ± 1.26 MPa of that of pure PCL scaffolds to 5.02 ± 1.68 MPa (PCL/PPy 0.5%) and 4.82 ± 0.84 MPa (PCL/PPy 1%), which is closer to the properties of the native human peripheral nerve (~6.5 MPa) (Dumont and Born, 2005; Nectow et al., 2011). PCL/PPy 2% scaffolds are much weaker than the other scaffolds, which might be due to the viscosity changes due to higher concentration of PPy-b-PCL. NGCs made of PCL/PPy thus could withstand the biological stresses when implanted inside the body.

The degradability of the PCL/PPy scaffolds were assessed by accelerated *in vitro* degradation studies using 0.5 M NaOH solution. Degradation studies, if carried out in physiological conditions, might take several months for evaluating the degradation profiles of slow degrading polymers such as PCL. In such cases, accelerated degradation studies are carried out to simulate similar degradation profiles within a short period of time (Lam et al., 2008). Degradation rate of PCL/PPy scaffolds are higher compared to the pure PCL scaffolds, while the scaffolds with the highest PPy-b-PCL concentration having a greater weight loss (Figure 7). Although the stress-strain curves of all the scaffolds follow a characteristic stress-strain curve of polymers at the start of the study, the behavior is lost with the passage of time as the scaffolds degrade and disintegrate. Mechanical testing results at different degradation time points (Table 2), indicates that the scaffolds start losing their mechanical integrity rapidly starting day 7 and the scaffold begins to disintegrate. It is to be noted that even with such harsh environment, the scaffolds withstand the stress till day 7, having appreciable mechanical properties. Given that the physiological conditions are much milder than the testing conditions, the NGCs could maintain their mechanical integrity much longer than 7 days in *in vivo* conditions.

As a proof-of-concept, in this study we are primarily interested to understand the impact of the conductive scaffolds on promoting peripheral neuronal growth. In our previous study using PCL scaffolds (Vijayavenkataraman et al., 2018), we had used PC12 cells cultured under neural differentiation conditions as representative of neural cells. However, the PC12 cells is a cell line derived from a tumor (pheochromocytoma) of the rat adrenal medulla which means these cells are not physiologically normal, though neural crest-derived they are not neural cells *per se* and lastly, they are of animal origin. PC12 cells and neuronal cells derived from rat dorsal root ganglia are often used as representative of peripheral neuronal cells as it is not possible to culture human-derived peripheral neuronal cells. We had earlier reported a methodology to derive peripheral neurons using hESCs-derived NCSCs as source of cells (Zhu et al., 2017). The peripheral neuronal cells differentiated from hESC-NCSCs have the advantage of being human-derived, representative of peripheral neurons and are physiologically normal (not tumor-derived). Hence, in this study, we used hESC-NCSCs to investigate the impact of PPy-b-PCL based conductive scaffolds on the growth and differentiation to peripheral neuronal cells. The results demonstrate that the hESC-NCSCs were able to grow on PCL and PCL/PPy scaffolds, in particular the PCL/PPy (1 and 2%) scaffolds supported higher growth of neural cells as demonstrated by the higher proliferation, and higher normalized fluorescence intensity. β3 tubulin is a pan-neuronal marker expressed among all neuronal lineages. NF-H is a subunit of neurofilament proteins that is expressed within peripheral neurons and in particular, is more strongly expressed in mature peripheral neurons (Yuan et al., 2017). Hence, the expression of NF-H is indicative of maturation status of neurons. Hence, the PCL/PPy scaffolds (in particular, PCL-PPy 1%) supported stronger maturation of hESC-NCSCs to peripheral neuronal cells as demonstrated by higher expression of *PRPH* and *NEFH* transcripts and higher volumetric fluorescence intensity of NF-H staining. Overall, these results suggest that PCL/PPy 1% based conductive scaffolds better supports and enhances the differentiation and maturation of hESC-NCSCs to peripheral neurons and hence, have potential clinical value as cell-free or cell-laden NGCs for peripheral neuronal regeneration.

The use of scaffold materials that conduct electricity have been shown to be advantageous for the growth of neurons as these cells have an inherent capacity to be excited by electricity (Mammadov et al., 2013). Previous studies on electrospun poly-L-lactic acid with carbon nanotubes have shown that increased electrical conductivity promotes overall neural differentiation of mouse embryonic stem cells (Mammadov et al., 2013). In another work, poly(L-lactide-co-ε-caprolactone) was combined with a conductive polymer, polyaniline to create a scaffold material that supported the growth of neurites in PC-12 cell lines (Bhang et al., 2012). In our study, addition of PPy to PCL increased electrical conductive properties of the scaffold material progressively from 0.28 ± 0.02 mS/cm in PCL/PPy 0.5%, to 1.15 ± 0.03 mS/cm in PCL/PPy 2%. The culture and differentiation of hESC-NCSCs on various PCL/PPy scaffolds showed that peripheral neural lineage cell types preferred PCL/PPy 1% which had a conductive property of 1.02 ± 0.03 mS/cm. Though, as to why the differentiation was better with PCL/PPy1% and not with PCL/PPy2% which has higher conductance, requires further speculation. Though the PCL/PPy scaffolds have conductive properties, electrical stimulus is not provided in this study. So, future studies on the impact of electrical stimulus and conductive scaffolds on the growth, maturation and alignment of peripheral neuronal cells will provide more insights on the regenerative potential of the PCL/PPy-based conductive NGCs. Further, future studies in animal models of peripheral neuron damage would help to validate the regenerative potential of these conductive polymer-based NGCs.

## Conclusion

Conductive and biodegradable PCL/PPy scaffolds with three different concentrations of PPy-b-PCL (0.5, 1, and 2% v/v) fabricated using EHD-jet 3D Printing method were evaluated for their potential as NGCs for peripheral nerve regeneration. Inclusion of PPy-b-PCL into PCL-based scaffolds aids the fabrication of softer scaffolds with conductive properties, mechanical properties similar to native human peripheral nerve (~6.5 MPa), improved degradation profiles and ability of aid growth and differentiation of peripheral neuronal cells *in vitro*. Overall, our results suggest that PCL/PPy scaffolds may be a promising material for guidance conduits in nerve tissue regeneration. The use of neural stem cells on electro-conductive scaffolds produces a symbiotic combination that may have high potential in future for the treatment of neurodegenerative disorders.

## Data Availability Statement

All datasets generated for this study are included in the manuscript/Supplementary Files.

## Author Contributions

SV conceived and designed the experiments, analyzed the data, and wrote the paper. SV and SK performed the experiments. SV and GS reviewed and edited the paper. SV, GS, TC, JF, and WL worked on funding acquisition. GS, TC, JF, and WL supervised the whole work.

### Conflict of Interest

The authors declare that the research was conducted in the absence of any commercial or financial relationships that could be construed as a potential conflict of interest.
