# Supplementary material for: 3D-Printed PCL/PPy Conductive Scaffolds as Three-Dimensional Porous Nerve Guide Conduits (NGCs) for Peripheral Nerve Injury Repair
Source: Front Bioeng Biotechnol. 2019 Oct 16;7:266. doi: 10.3389/fbioe.2019.00266 (PMC6843025; doi:10.3389/fbioe.2019.00266)
Supplement: Supplementary file 1 [file Table_1.DOCX]

**Supplementary Information**

**3D-Printed PCL/PPy Conductive Scaffolds as Three-dimensional Porous Nerve Guide Conduits (NGCs) for Peripheral Nerve Injury Repair**

Sanjairaj Vijayavenkataraman^1^, Sathya Kannan^2^, Cao Tong^2^, Jerry Y.H. Fuh^1^, Gopu Sriram^2^, Wen Feng Lu^1^

^1^ Department of Mechanical Engineering, National University of Singapore (NUS), Singapore

^2^ Faculty of Dentistry, National University of Singapore (NUS), Singapore

**SUPPLEMENTARY METHODS**

**Image analysis**

Imaris version 9.2.1 software was used for quantifying fluorescence intensity. Under the surpass view, the surface tool was used which creates a semiautomatic 3D surface on the image (***Supplementary Figure 1***). Initial thresholding was done, and these levels were kept constant for each channel and the complete data set. Following surface creation, volume of the surface created and its mean fluorescence intensity is automatically generated by the software. As images are 3D z-stacks, volumetric data was taken for the calculations. Mean fluorescent intensity values of ꞵ3 tubulin (green channel) and NF-H (red channel) were measured, from which the fluorescence was normalized to ꞵ3 tubulin expression to obtain the normalized fluorescence intensity (***Figure 9B***). Then, volume of expression for these two proteins were normalized to the total cell numbers present in each region of interest (ROI) to plot the normalized fluorescence volume (***Figure 9C***).

**SUPPLEMENTARY TABLES**

**Supplementary Table 1. Details of RT-PCT primers**

| **Gene** | **Sequence F** | **Sequence R** |
| --- | --- | --- |
| *HNK1* | CTCCTTCGAGAACTTGTCACC | GGGTCAGTGAAGCCCTTCTT |
| *TUBB3* | GGCCAAGGGTCACTACACG | GCAGTCGCAGTTTTCACACTC |
| *PRPH* | GCCTGGAACTAGAGCGCAAG | CCTCGCACGTTAGACTCTGG |
| *NEFH* | TGAACACAGACGCTATGCGCTCAG | CACCTTTATGTGAGTGGACACAGA |
| *GAPDH* | GGAGCGAGATCCCTCCAAAAT | GGCTGTTGTCATACTTCTCATGG |

**SUPPLEMENTARY FIGURES**


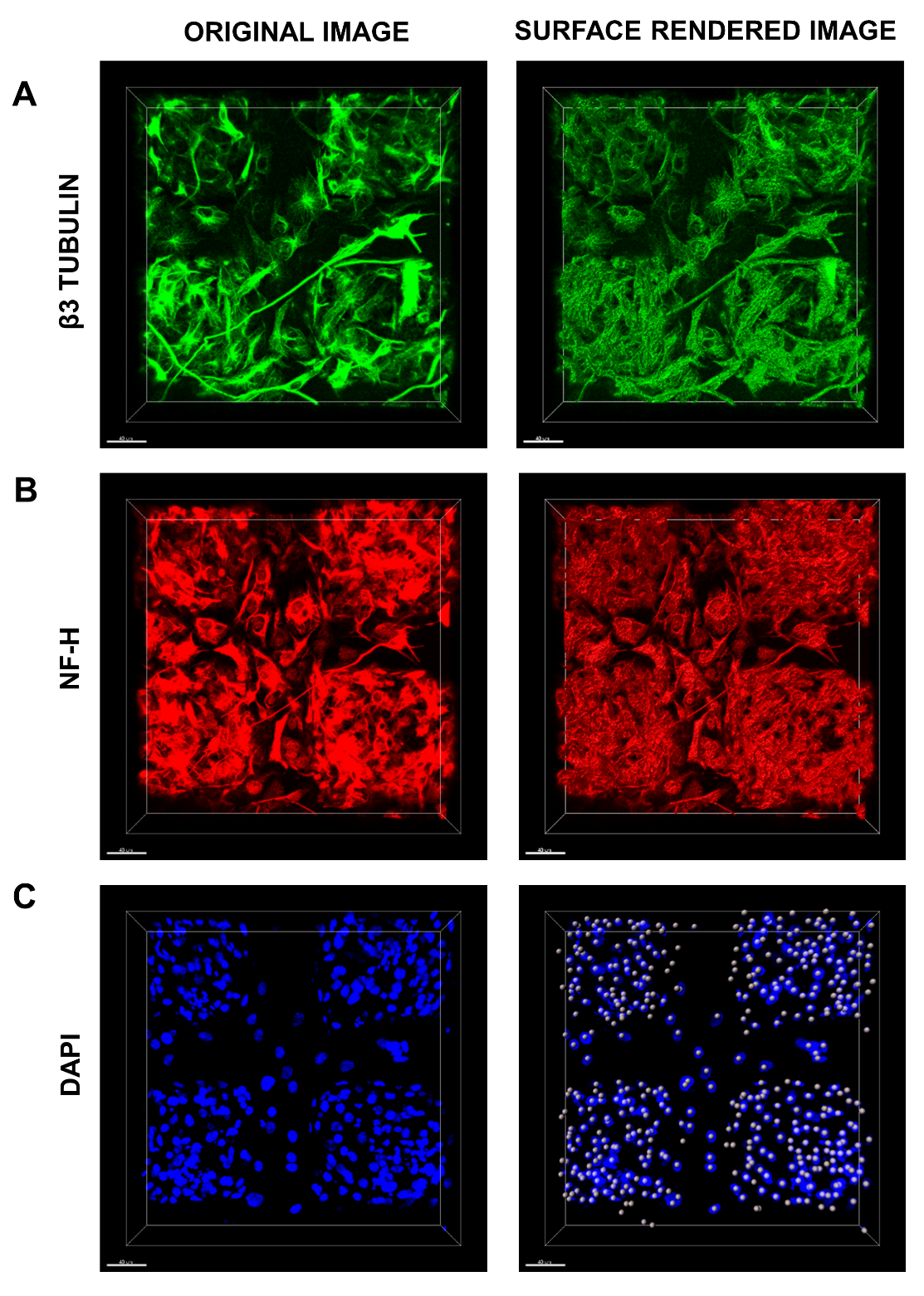


**Supplementary Figure 1. Image analysis.** A. Green channel images showing expression of ꞵ3 tubulin (left) and volumetric surface creation (right). B. Red channel showing expression of NFH (left) and volumetric surface expression (right). Blue channel showing nuclei of cells (left) and counting with spots (right).
